# Accelerated real time 2D and segmented 3D cine imaging – whole heart approaches in a single breath hold

**DOI:** 10.1186/1532-429X-11-S1-P225

**Published:** 2009-01-28

**Authors:** Yu-Po Chen, Sven Zuehlsdorff, Peter Weale, John Sheehan, Renate Jerecic, James C Carr

**Affiliations:** 1 Northwestern University Feinberg School of Medicine, Chicago, IL USA; 2 Siemens Medical Solutions USA, Inc., Chicago, IL USA

**Keywords:** Left Ventricle, Single Breath Hold, Left Ventricle Volume, Measured Ejection Fraction, Prospective Trigger

## Introduction

The clinically well-established technique used to assess global functional cardiac parameters such as ejection fraction (EF) is a 2D cine approach in which the left ventricle (LV) is covered in multiple breath holds. The development of 32 channel receiver coils allows using high parallel acceleration factors with and increased signal to noise ratio (SNR). In this volunteer study, single breath-hold approaches have been used and the derived functional parameters (ejection fraction EF, end diastolic volume EDV, end systolic volume ESV, stroke volume SV, and myocardial mass MM) have been compared to the clinical standard technique. There is a good correlation between the standard and the single breath-hold techniques. They can potentially substitute conventional multi-breath hold approaches to increase patient comfort and reduce the study time.

## Materials and methods

A prospectively gated multi-slice 2D real-time cine sequence was implemented on a 1.5 T scanner (MAGNETOM Espree, Siemens AG Healthcare Sector, Erlangen, Germany) using highly accelerated acquisition schemes. For parallel imaging, coil reference data have been derived using previously acquired reference data or a temporal method (TGRAPPA [1]), respectively. Optional, outer *k*-space data could be shared between consecutive phases to increase temporal resolution. For a segmented 3D cine approach, a TGRAPPA acquisition scheme in phase and partition encoding direction was used. Both 2D and 3D approaches allowed to acquire cine data covering the entire LV in one single breath hold using typical parameters shown in Table 1. Cine data of ten healthy volunteers (2 male, 8 female; mean age 27.5; range 21–40) have been acquired using a 32-channel cardiac coil (Invivo, Orlando, Florida) and the derived functional parameters have been compared using a retrospectively gated multi-breath hold protocol as clinical standard.

**Table 1:** Protocol parameters

	2D Segmented (GRAPPA)	2D (GRAPPA)	2D (TGRAPPA)	3D (TGRAPPA)
TR/TE (ms)	2.84/1.2	2.4/1.04	2.3/1	2.9/1.44
Temporal Resolution (ms)	29.9 (interpolated)	45.8	46.6	51.84
FOV	340 × 276	340 × 289	340 × 288	380 × 306
Slice/Partition Thickness (mm)	8	8	7	6
Voxel Size (mm^3^)	2.5 × 1.8 × 8.0	2.3 × 2.1 × 8.0	3.6 × 2.4 × 7.0	3.1 × 2.6 × 6.0
Acceleration Factor	GRAPPA 2 (Integrated Reference Lines)	GRAPPA 4 (separate reference lines and shared phases)	TGRAPPA 2 × 2	TGRAPPA 2 × 2 (Non-isotropic)
Triggering	Retrospective	Prospective	Prospective	Prospective
Number of Breath holds	10	1	1	1

Functional analysis was performed using Argus (Siemens AG Healthcare Sector). Volumetric analysis was performed on all cine images; EF, EDV, ESV, SV, and MM were derived. Paired Student's t-test and linear regression were performed. A level of p < 0.05 was considered statistically significant.

## Results

Linear regression showed good correlation (r > 0.75) between the measurements of LV function parameters yielded by the standard segmented 2D protocol and those by the experimental protocols, except in measuring EF and SV.

The same analysis also showed strong correlation among the single breath-hold techniques in measuring LV parameters.

Paired t-tests indicated that there were no significant differences in measured EF and MM between the described protocols. However, significant differences were found in EDV, ESV and SV values; segmented 2D protocol consistently yielded higher values than the other protocols in these cases.

## Discussion

The results of linear regression and paired t-tests suggest that the real-time 2D TGRAPPA technique can potentially substitute the standard clinical protocol. However, the data showed that the experimental protocols tended to underestimate EDV, ESV, and SV. It has been reported that prospective triggering leads to significantly lower EDV and SV values, since the late diastole is not covered by prospective triggering [2]. Since the gold standard in this study is retrospectively triggered, the difference in ECG triggering could explain the significant differences in LV volumes.

**Table 2:** Correlation (*r*) of LV function parameters between the standard and each of the single breath-hold techniques

	2D (GRAPPA)	2D (TGRAPPA)	3D (TGRAPPA)
EF	0.70	0.60	0.64
EDV	0.75	0.88	0.76
ESV	0.85	0.88	0.79
SV	0.38	0.62	0.59
MM	0.93	0.74	0.83

**Table 3:** Correlation (*r*) in measurements between pairs of the single breath-hold techniques

	2D (GRAPPA)/2D (TGRAPPA)	2D (GRAPPA)/3D (TGRAPPA)	2D (TGRAPPA)/3D (TGRAPPA))
EF	0.86	0.89	0.93
EDV	0.90	0.92	0.90
ESV	0.89	0.90	0.93
SV	0.89	0.89	0.85
MM	0.86	0.90	0.74

**Table 4:** Paired T-tests between the standard and each of the single breath-hold techniques (NS = no significant difference)

	2D (GRAPPA)	2D (TGRAPPA)	3D (TGRAPPA)
EF	0.54(NS)	0.98 (NS)	0.72 (NS)
EDV	P < .05	P < .05	P < .01
ESV	P < .05	0.06(NS)	P < .05
SV	P < .05	0.06(NS)	P < .01
MM	0.98(NS)	0.08(NS)	0.26(NS)
